# O7.6. META-ANALYSIS OF EFFICACY OF COGNITIVE ENHANCERS FOR PATIENTS WITH SCHIZOPHRENIA-SPECTRUM DISORDERS

**DOI:** 10.1093/schbul/sby015.235

**Published:** 2018-04-01

**Authors:** Igne Sinkeviciute, Marieke Begemann, Merel Prikken, Bob Oranje, Erik Johnsen, Wan U Lei, Kenneth Hugdahl, Rune A Kroken, Carina Rau, Jolien Jacobs, Silvia Mattaroccia, Iris E Sommer

**Affiliations:** 1 Haukeland University Hospital; 2 University Medical Centre Utrecht, Brain Centre Rudolf Magnus, University Medical Center Groningen; 3 University Medical Centre Utrecht, Brain Centre Rudolf Magnus; 4 Haukeland University Hospital, NORMENT Center of Excellence, University of Oslo, University of Bergen; 5 School of Medicine, Medical Sciences and Nutrition, University of Aberdeen; 6 University of Bergen; Norwegian Center of Excellence for Mental Disorders Research, University of Oslo; Haukeland University Hospital; 7 University of Konstanz; 8 Utrecht University; 9 University of Rome; 10 University of Groningen, University of Bergen

## Abstract

**Background:**

Cognitive impairment is a core feature of schizophrenia, which is predictive for functional outcomes and should therefore be one of the main treatment targets. Pharmacological enhancement of cognition has been a main field of research in the last decades, investigating almost all neurotransmitter systems and reporting positive as well as negative findings. The pathophysiology of cognitive dysfunctions in schizophrenia is complex and many different neurotransmitter systems are involved. This quantitative review provides an overview of studies of pharmacological agents targeting neurotransmitter systems relevant for cognitive deficits in schizophrenia.

**Methods:**

In our systematic search we included pharmacological agents targeting glutamatergic, cholinergic, serotonergic, dopaminergic, GABAergic and noradrenergic neurotransmitter systems, and also a miscellaneous group of agents, including modafinil/armodafinil. We evaluated the effects of cognitive enhancers on overall cognitive functioning as well as on seven cognitive domains, including attention/vigilance, processing speed, reasoning, verbal learning and memory, visual learning and memory, working memory, and verbal fluency.

**Results:**

In total, 93 studies with 5630 patients were suitable for inclusion in the meta-analysis. The mean sample size was 28.73 (SD=27.13, range=4–203), mean age of the participants was 44.15 years (SD=6.36, as reported by 91 study samples), 68.54% of the sample were men (as reported by 87 study samples), and average illness duration was 15.57 years (SD=6.47, as reported by 63 study samples). Combining all cognitive enhancers across different neurotransmitter systems for the effect on overall cognitive functioning resulted in fifty-one study samples, with a total of 3635 patients. Cognitive enhancers showed a small but significant positive effect size of 0.10 over placebo treatment (p=.023; 95%CI=0.01 to 0.18). Overall, cognitive enhancers showed no positive effects as compared to placebo for the separate domains. When analysing each neurotransmitter system separately, agents acting predominantly on the glutamatergic system showed a small but significant effect size on overall cognitive functioning (Hedges’ g=0.19, p=.01), as well as on working memory (Hedges’ g=0.13, p=.04). A sub-analysis of acetylcholinesterase inhibitors (AChEI) within cholinergic system showed a small effect on working memory (Hedges’ g=0.26, p=.03). No other positive effects of cognitive enhancers as compared to placebo were revealed.

**Discussion:**

The current meta-analysis showed very few favorable effects of cognitive enhancers for patients with schizophrenia spectrum disorders. The overall analysis showed small difference between cognitive enhancers and placebo. Most studies were on agents acting on the glutamatergic and the cholinergic system. There is some evidence of positive effects on cognitive functioning for agents acting on glutamatergic system and acetylcholinesterase inhibitors within cholinergic system. There is still a major lack of studies involving agents acting on other systems. Important issues such as dose, treatment duration, including a younger population and subtyping heterogeneous samples should be taken into account for future studies.

